# CD147 Levels in Blood and Adipose Tissues Correlate with Vascular Dysfunction in Obese Diabetic Adults

**DOI:** 10.3390/jcdd9010007

**Published:** 2021-12-28

**Authors:** Mohamed M. Ali, Imaduddin Mirza, Dina Naquiallah, Chandra Hassan, Mario Masrur, Francesco M. Bianco, Abeer M. Mahmoud

**Affiliations:** 1 Department of Physical Therapy, College of Applied Health Sciences, University of Illinois at Chicago, Chicago, IL 60612, USA; mali37@uic.edu; 2 Division of Endocrinology, Diabetes, and Metabolism, Department of Medicine, College of Medicine, University of Illinois at Chicago, Chicago, IL 60612, USA; mmirza24@uic.edu (I.M.); dnaqui2@uic.edu (D.N.); 3 Department of Surgery, College of Medicine, University of Illinois at Chicago, Chicago, IL 60612, USA; chandrar@uic.edu (C.H.); mmasrur@uic.edu (M.M.); biancofm@uic.edu (F.M.B.)

**Keywords:** obesity, diabetes, vascular dysfunction, CD147, matrix metalloproteinases, glycosylation, advanced glycation end-products, visceral adipose tissue, cardiometabolic risk

## Abstract

CD147 is a glycoprotein that stimulates the production of matrix metalloproteinases (MMPs), known contributors to cardiovascular risk. The activity of CD147 protein depends on its glycosylation. However, it is unclear whether CD147 protein expression or glycosylation are influenced by the diabetic milieu characterized by hyperglycemia and abundant glycation-end-products (AGEs). We examined the circulating and visceral adipose tissue (VAT) levels of CD147 and their correlation with vascular function in obese, obese diabetic, and non-obese controls (*n* = 40, each). The circulating levels of CD147 and the glycosylated CD147 protein in VAT were considerably higher in obese, particularly obese diabetic subjects compared to controls. Obese diabetics had the lowest brachial and arteriolar vasoreactivity and the highest carotid pulse-wave velocity (PWV, a measure of arterial stiffness) among the three groups. CD147 correlated positively with body mass index (BMI), total and visceral fat mass, PWV, and plasma levels of glucose, insulin, MMPs, and AGEs and negatively with brachial artery and VAT-arteriolar vasoreactivity and nitric oxide production. Multivariate regression revealed that BMI, body fat mass, insulin, and glucose levels significantly predicted CD147. Our data suggest that higher levels of CD147 in obese subjects, particularly those with diabetes, are linked to vascular dysfunction and several cardiometabolic risk factors.

## 1. Introduction

Diabetes is a global epidemic with a growing healthcare impact. There is a well-established link between diabetes and an increased risk of developing cardiovascular disease (CVD) [1]; however, significant pathophysiological mechanisms have yet to be identified. Previous studies have reported a contributing role of matrix metalloproteinases (MMPs) to the development of hypertension and CVDs such as atherosclerosis [2]. The expression and activity of these extracellular remodeling enzymes have been shown to increase in atherosclerotic plaques [3] and in patients with hypertension, coronary artery diseases, and myocardial infarction [2]. Accordingly, the proteins that regulate MMPs are prospective therapeutic targets with the potential to lower cardiovascular risk and need further exploration. CD147, also known as extracellular matrix metalloproteinase inducer (EMMPRIN), is one of the essential MMP regulators, and it is critical to determine its potential role in the association between metabolic diseases and vascular dysfunction [4].

CD147 is a transmembrane glycoprotein that participates in various physiological processes and is mainly active when glycosylated [5]. Variations in CD147 glycosylation has been discovered to influence its function. MMP activity has been observed to be induced primarily by the highly glycosylated version of CD147. [5]. Aberrant glycosylation of CD147 was implicated in several pathological conditions such as atherosclerosis [6], liver cirrhosis [7], and cancer invasion and metastasis [8]. In most of these conditions, concomitant upregulations of MMP activity were reported [9,10]. However, there is a lack of comparable reporting of CD147 status in obesity and diabetes. Glycoprotein formation occurs secondary to an enzymatic attachment of sugar to proteins as a part of the posttranslational protein modifications. This binding could also happen non-enzymatically as found in glycated hemoglobin, glycated albumin, and AGEs. Both forms of protein glycosylation have been proven to be induced by the abundance of sugar substrate [11]. 

We recently demonstrated inductions in CD147 expression and enzymatic glycosylation and MMP2/9 activity in adipocytes treated with high glucose and AGEs [12]. Furthermore, we observed concomitant upregulations of the N-acetylglucosaminyltransferase, MGAT5, which induces CD147 glycosylation. These effects were reversed by the glycosylation inhibitor, tunicamycin, and inhibitors of RAGE (receptor of AGEs) and NFκB (nuclear factor κ B). Similarly, a prior study reported an increased MMP expression and activity in monocytes incubated with high glucose levels [13]. The mechanism underlying this observation was considered to be related to CD147 upregulation. Chronic hyperglycemia in diabetes promotes the formation of AGEs, which have been linked to a number of diabetes-related complications including vascular dysfunction and organ damage [14]. We previously reported elevated levels of AGEs and their receptor, RAGE, in diabetic patients’ tissues compared to healthy controls [15]. Nevertheless, no clinical evidence has been found to relate these findings to CD147 expression, glycosylation, and downstream MMP induction. Thus, in the current study, we sought to explore circulating CD147 levels as well as its protein expression and glycosylation in the adipose tissues of obese and obese diabetic subjects compared to healthy controls. Moreover, we tested the correlation of these levels with in vivo measured arterial function, ex vivo measured arteriolar vasoreactivity, and several other cardiometabolic risk factors.

## 2. Materials and Methods

### 2.1. Study Participants

Participants in the study include obese, obese-diabetic, and healthy controls (*n* = 40, each) who had bariatric (obese group) or elective surgeries (non-obese) at the University of Illinois Hospital. The inclusion standards included adults less than 50 years old with a body mass index <25 kg/m^2^ for the non-obese controls and >35 kg/m^2^ for the obese participants. Current use of physician confirmed diabetes medication and fasting glucose ≥126 mg/dL was used as the criteria for T2D. Pregnancy, current smoking, a history of previous bariatric surgery, current chronic inflammatory and autoimmune conditions, heart disease, liver disease, or kidney diseases were all exclusion factors. Finally, any severe chronic disease or inflammatory illness that could influence vascular outcomes was ruled out. Subjects were evaluated for eligibility criteria before surgery during a data collection clinical visit to the University of Illinois Clinical Research Core. The study’s specifics were explained to eligible subjects, and those who chose to participate gave written informed consent. Blood samples, vital signs (heart rate, blood pressure, and respiratory rate), anthropometric/body composition measurements (via DEXA scan), brachial artery ultrasound imaging, and pulse wave velocity (PWV) measurements were obtained. Visceral adipose tissue samples were acquired during the bariatric/elective surgeries and instantly put in a cold HEPES (4-(2-hydroxyethyl)-1-piperazineethanesulfonic acid) buffer solution for tissue dissection and microvessel isolation. The current study’s techniques and procedures were approved by the University of Illinois Institutional Review Board and met the requirements established by the Declaration of Helsinki’s most recent iteration.

### 2.2. Anthropometric and Cardiometabolic Measures

The following variables were evaluated: age, gender, body weight, waist circumference, and BMI. To determine the total body and visceral adipose tissue mass versus lean mass, we used DEXA (Dual X-ray absorptiometry) scanning (iDXA, General Electric Inc., Boston, MA, USA). All individuals underwent a single DEXA scan in accordance with the manufacturer’s handbook. Biochemical parameters were measured in fasting blood samples (lipid profile and glucose metabolism). A standard glucometer was used to assess plasma glucose levels (LifeScan, Milpitas, CA, USA). Following the manufacturer’s protocol, plasma insulin was tested using sensitive ELISA kits (Enzo Life Sciences, Inc., Farmingdale, NY, USA). As previously described, the homeostasis model assessment for insulin resistance (HOMA-IR) was computed using the formula: fasting insulin (µU/L) × fasting glucose (nmol/L)/22.5 [16]. The lipid profile including total cholesterol, triglycerides, high-density lipoproteins (HDL), and low-density lipoproteins (LDL) were evaluated via enzymatic assays using a Hitachi 911 analyzer and reagents from Roche Diagnostics (Indianapolis, IN, USA). 

### 2.3. Plasma CD147, MMP2/9, and AGEs

Plasma CD147, MMP2, and MMP9 levels were determined using Human EMMPRIN/CD147, MMP2, and MMP9 Quantikine ELISA assays (R&D Systems, McKinley Place NE, MN, USA), respectively. Briefly, 2× diluted plasma samples were prepared and incubated in the corresponding protein conjugate (CD147, MMP2, or MMP9) coated plate for 2 h on a shaker at room temperature. This was followed with 30-min incubation with the corresponding substrate solution, and the reaction was terminated by the provided stop solution. The absorbance was then assessed immediately on an iMark Absorbance plate reader (BioRad Laboratories, Hercules, CA, USA) at a primary wavelength of 450 nm. Plasma levels of AGEs mainly carboxymethyl lysine (CML) and methylglyoxal (MG) were measured using specific ELISA kits (BioVision Inc., Milpitas, CA, USA). Plasma samples were incubated with a biotin-labeled specific antibody working solution for 45 min at 37 °C followed by incubation with HRP-streptavidin conjugate for 30 min. For color development, incubation with tetramethylbenzidine (TMB) substrate was performed at 37 °C protected from light for 15–30 min. Lastly, the reaction was terminated using the stop solution, and the absorbance was assessed on a plate reader at 450 nm.

### 2.4. Nitric Oxide (NO) and Inflammatory Biomarkers

Nitrate and nitrite, stable NO metabolites, were quantified in plasma samples using the Griess-based reaction kit purchased from Cayman Chemicals (Ann Arbor, MI, USA), following our previously published protocol [17,18,19]. Briefly, samples were ultra-filtered via Millipore centrifugal filters with a cut-off molecular weight of 10 KDa (Burlington, MA, USA). Nitrate was converted into nitrite in filtered samples using the enzyme nitrate reductase. Plasma nitrites were converted into a dark purple azo molecule after the addition of Griess reagents. Microplate Reader (iMark, BioRad) was used to measure the absorbance at 540 nm. The concentration of nitrate was estimated based on the nitrate standard curve. An ultra-sensitive ELISA assays (Crystal Chem, Elk Grove Village, IL, USA) were utilized to assess plasma concentrations of C-reactive protein (CRP). Plasma samples and the provided controls and standards were incubated with the primary antibody for one hour before being incubated with the working HRP solution (60 min), followed by the substrate solution (20 min) and lastly, the stop solution to end the reaction. The absorbance was determined using a microplate reader at 450 nm. To quantify interleukin-6 (IL6) and tumor necrosis factor-alpha (TNFα) in plasma, High Sensitivity Luminex Assays (R&D Systems, McKinley Place NE, MN, USA) were used. Samples were incubated with magnetic microparticles coated with antibody and embedded with fluorophores. After removing non-specific binding with washing processes, analyte-specific biotinylated antibodies followed by streptavidin-phycoerythrin conjugate were added. Lastly, the microparticles were resuspended and analyzed with the Luminex MAGPIX plate reader (Thermo Fisher Scientific, Waltham, MA, USA).

### 2.5. In Vivo Vascular Measurements

Brachial flow-mediated dilation (FMD) was measured using Hitachi Prosound Alpha 7 (Hitachi Aloka Medical America, Wallingford, CT, USA). For recording, a linear probe was positioned five centimeters above the left arm’s antecubital fossa, and a 1-min baseline imaging (BSL) was recorded. Then, a blood pressure cuff was put around the mid-forearm and inflated to 220 mmHg for 5 min. Following cuff deflation (reactive hyperemia, RH), a video grabber was used to record a 300-s video sequence at three frames per second for offline measurement. For simultaneous measurement of blood flow velocity, a pulsed wave Doppler was utilized. The brachial artery diameter was measured using the Brachial Analyzer program (Medical Imaging Applications LLC, Coralville, IA, USA). The greatest baseline diameter of the brachial artery was deducted from the largest diameter obtained following the blood pressure cuff deflation to determine relative FMD [percent FMD = (RH diameter in mm-BSL diameter in mm / BSL diameter in mm) × 100]. The waveform at the carotid and femoral (central) sites was used to compute the central pulse wave velocity (PWV). All waveforms were detected on the right side of the body with applanation tonometry (SphygmoCor; AtCor Medical, Sydney, Australia). This approach is described in detail in previous publications by our research group [17,18,19,20]. 

### 2.6. Arteriolar FID and NO Measurements

Adipose tissue samples were dissected, and resistance arterioles were isolated then prepared for flow-induced dilation (FID) measurements in response to a gradual increase in intraluminal pressure gradient as we previously reported [17,18,19,21,22,23,24]. Briefly, the dissected arterioles were cannulated via glass micropipettes in an organ perfusion chamber and visualized by video microscopy. Arterioles were perfused with a heated salt solution (Krebs buffer) containing (in mmol/L): NaCl (123), CaCl_2_ (2.5), NaHCO_3_ (20), KCL (4.4), MgSO_4_ (1.2), KH_2_PO_4_ (1.2), and glucose. The buffer’s temperature was kept at 37 °C, and the pH of the solution was maintained at 7.4. Krebs buffer was provided with a mixture of O_2_ (21%), CO_2_ (5%), and N_2_ (74%). An intraluminal pressure gradient (10–100 cm H_2_O) was created by changing the distance between Krebs reservoirs connected to both ends of the arterioles. At baseline, cannulated arterioles were preconstructed with endothelin-1 (ET-1) (Sigma Aldrich, Burlington, MA, USA), and arteriolar vasodilation was reported as the percentage increase in diameter in response to increasing intraluminal pressure gradient relative to the endothelin-1-constricted state. The NO produced by isolated arterioles was quantified using the NO Detection Kit (Enzo Life Sciences, Farmingdale, NY, USA), as previously described [21,22,23]. NO was measured in cannulated arterioles in response to a physiological intra-arteriolar pressure gradient (Δ60 cm H_2_O). Arterioles were incubated with NO detection reagents then removed and mounted on microscope slides for image capturing via a fluorescence microscope (Eclipse TE 2000, Nikon, Japan) at 650 nm wavelength. 

### 2.7. Western Blotting

Total proteins were extracted from adipose tissue samples using a mixture of RIPA lysis buffer (Cell Signaling) and protease and phosphatase inhibitors (Sigma-Aldrich, Burlington, MA, USA). Proteins were quantified using BCA Protein Assays (Thermo Fisher Scientific). Total protein (10 µg) was gel electrophoresed and transferred to polyvinylidene fluoride (PVDF) membranes. PVDF membranes were then incubated overnight at 4 °C with the primary mouse monoclonal antibodies (CD147, MGAT3, MGAT4a, MGAT5, and loading control GAPDH; Abcam, Waltham, MA, USA), and then with infrared IRDye-labeled secondary antibodies (LI-COR Biosciences, Lincoln, NE, USA). Lastly, membranes were scanned with an infrared imaging system (Odyssey Clx; LI-COR Biosciences). The target protein band intensity was assessed relative to the loading control using Image Studio (LI-COR Biosciences).

### 2.8. MMP2/9 Activity Assay

The MMP2 and MMP9 activity in the isolated VAT protein was determined using the InnoZyme^TM^ Gelatinase (MMP2/9) Activity Assay (Millipore Sigma, Burlington, MA, USA). This approach is based on the FRET (fluorescence resonance energy transfer) principle. The FRET peptide which is highly selective for MMP2 and MMP9 emits fluorescence only when it is cleaved; hence, this peptide is utilized as a marker of MMP2 and MMP9 activity. In this experiment, isolated proteins were adjusted to equal concentrations. P-Aminophenylmercuric Acetate (APMA) was used to activate pro-MMP2/9 followed by incubation with the provided substrate for 6 h at 37 °C. The emitting fluorescence was then measured using a multimode plate reader (iMark Absorbance Microplate Reader; BioRad, Hercules, CA, USA) at 320 and 405 nm for excitation and emission, respectively.

### 2.9. Immunoprecipitation and Glycoprotein Staining

CD147 protein was immunoprecipitated from total proteins extracted from VAT samples using Dynabeads protein G magnetic beads (Life Technologies, Carlsbad, CA, USA). In this experiment, Dynabeads were primed with a CD147-specific monoclonal antibody (Abcam, ab108308) for 60 min at room temperature then incubated with total proteins extracted from VAT (200 µg) overnight at 4 °C. Immunoprecipitated CD147 proteins were extracted from the antigen-antibody Dynabead complex via SDS-PAGE sample buffer, then gel electrophoresed and labelled with the Pierce^TM^ Glycoprotein Staining Kit (Thermo Fisher Scientific). After a 30-min incubation in 50% methanol, gels were immersed for 0 min in 3% acetic acid, washed in distilled water, and incubated for 15 min with Oxidizing Solution (25 mL). This was followed by incubation in Glycoprotein Stain (25 mL) with gentle agitation for 15 min and in Reducing Solution (25 mL) for 5 min. Lastly, the gels were extensively rinsed with acetic acid (3%) followed by distilled water. The glycosylated CD147 protein were visible as pink bands that were scanned and quantified using the NIH ImageJ program.

### 2.10. Statistical Analyses

All continuous variables were reported as the average ± standard error (SE), and *p* < 0.05 was considered to be statistically significant. Analysis of variance (ANOVA) or Kruskal-Wallis Test, followed by appropriate post hoc tests, were used for between-group comparisons where variables followed or did not follow normal distribution, respectively. A bivariate Pearson Correlation was used to test for a statistically significant linear relationship between continuous variables. To evaluate the association between plasma CD147 levels and cardiometabolic risk factors, multiple regression analysis was used. In this model, we included BMI, body fat mass, VAT mass, plasma glucose, plasma insulin, and HbA1c. A multiple regression analysis that included blood pressure, brachial artery FMD, PWV, and arteriolar FID and NO was used to test the association between plasma CD147 and vascular function. Corrections for multiple testing was performed using Bonferroni correction. Statistical Analyses were made using SPSS statistical software (version 28.0; SPSS Inc., Chicago, IL, USA).

## 3. Results

### 3.1. Cardiometabolic Risk Factors

Physical characteristics of the study subjects and measured cardiometabolic risk factors were summarized in Table 1. Age was not statistically different among the study groups. Bodyweight, waist circumference, and body mass index (BMI) were significantly higher in the obese group compared to the non-obese controls. However, no significant differences were detected between the obese and obese-T2D groups. Similar trends were observed for total body and visceral fat mass measured via DEXA scanning. Obese subjects had higher systolic and diastolic blood pressure than controls, but only diastolic blood pressure was considerably higher in obese-T2D than non-diabetic obese subjects. The average levels of fasting plasma glucose (FPG) and HbAlc (glycosylated hemoglobin) in the obese non-diabetic and control groups were not statistically different. These measures, however, were notably higher in the obese T2D group. On the other hand, fasting plasma insulin (FPI) and the Homeostatic Model Assessment for Insulin Resistance (HOMA-IR) differed considerably among the three groups, with the highest levels observed in obese T2D participants. In terms of lipid profiles, obese-T2D participants had the highest total cholesterol, LDL, and triglycerides, whereas HDL values were much lower in this group. Plasma concentrations of the vasoactive mediator, nitric oxide (NO), were 47% and 76% lower in the obese and obese-T2D groups, respectively, compared to controls. Finally, obese participants had higher levels of the inflammatory markers C-reactive protein (CRP), IL6, and TNFα, which were significantly greater in obese-T2D subjects.

### 3.2. CD147, MMP2/9, and AGE Measurements

Plasma levels of CD147, MMP2, MMP9, methylglyoxal (MG), and carboxymethyl lysine (CML) were measured in all study subjects and reported in Table 1. Despite the significant inter-individual variations, the obese-T2D group had a significantly higher CD147 average than controls and non-diabetic obese (110% and 52% higher, respectively). Similar patterns were identified for both MG and CML; obese T2D had 134% and 75% greater MG and 160% and 51% higher CML than controls and obese non-diabetic, respectively. MMP2 and MMP9 concentrations, on the other hand, were higher in obese individuals compared to controls, with no significant difference between obese T2D and obese non-diabetic participants. 

The intracellular protein of CD147 exists in two forms, low glycosylated (LG; MW ~ 27 kDa) and high glycosylated (HG; MW ~ 60 kDa). In prior mechanistic investigations, we observed increases in enzymatic CD147 glycosylation in response to high glucose and carboxymethyl lysine (CML) treatments in cultured human adipocytes [12]. These inductions were associated with an increased activity of MMP2 and MMP9. Furthermore, an increase in MGAT5, an enzyme demonstrated to play a critical role in CD147 glycosylation, was also detected. In light of these findings, we sought to investigate CD147 glycosylation in the VAT collected from the study participants. We also measured the protein levels of three glycosyltransferases (MGAT3, MGAT4a, and MGAT5) previously reported to correlate with CD147 glycosylation in clinical samples such as cancer tissues. Furthermore, we aimed to see if these data were related to MMP2/9 activity measured in VAT-extracted proteins using gelatinase fluorogenic activity assay. 

**Figure 1:**
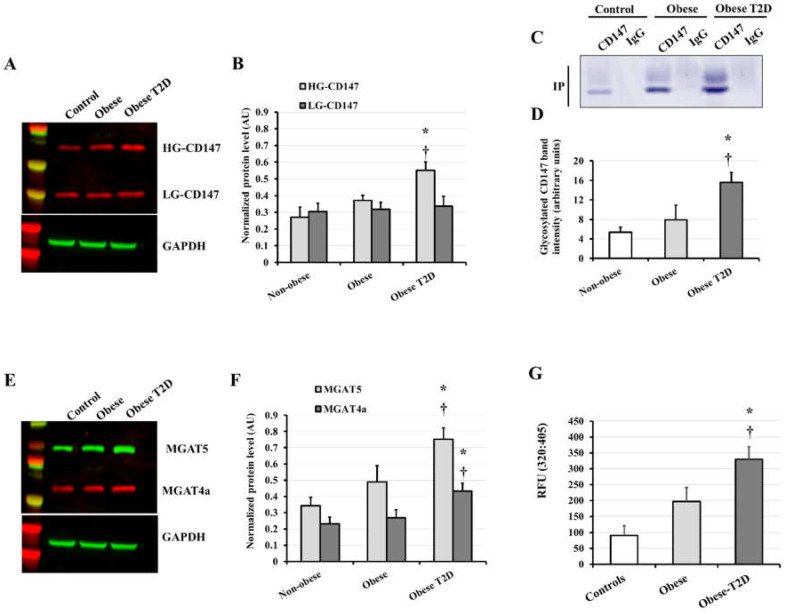
CD147 protein glycosylation and MMP activity in VAT. Representative image of Western blot analysis (**A**) and quantification of the normalized protein levels (**B**) of HG-CD147 and LG-CD147 proteins in VAT samples obtained from obese, obese-T2D, and control subjects. Representative image (**C**) and quantification (**D**) of glycosylated CD147 protein in the study subjects using Pierce™ Glycoprotein Stain. Representative Western blot (**E**) and quantification of the normalized protein levels (**F**) of MGAT5 and MGAT4a proteins in VAT biopsies acquired from obese, obese T2D, and control subjects. (**G**) Quantitative measurement of MMP2/9 activity using InnoZyme™ Gelatinase (MMP-2/MMP-9) Fluorogenic Activity Assay. Results represent the means ± standard error (SE). The * symbol indicates a *p*-value < 0.05 when comparing obese subjects with controls. The † symbol indicates a *p*-value < 0.05 when comparing obese and obese T2D subjects.

Our findings revealed that obese-T2D patients had the greatest amounts of the HG-CD147 form, with 49% (*p* = 0.025) and 103% (*p* < 0.001) greater levels than obese and non-obese controls, respectively (Figure 1A,B). No significant differences were observed in the LG-CD147 protein among the three groups. We further examined CD147 glycosylation by immunoprecipitating the protein from VAT-isolated proteins and gel electrophoresed it followed by staining the glycosylated protein fraction with periodic acid-Schiff (PAS) stain. Our data demonstrated greater levels of CD147 glycosylation in obese T2D subjects compared to other groups (~1-fold (*p* = 0.036) and 1.9 folds (*p* = 0.003) higher than obese non-diabetic subjects and controls, respectively) (Figure 1C,D). Regarding the glycosyltransferase MGAT3, no statistically significant differences were found among the three groups (data not shown). MGAT4a and MGAT5, on the other hand were significantly greater in the obese T2D group compared to obese non-diabetic (MGAT4a: 61%, *p* = 0.039; MGAT5: 54%, *p* = 0.042) and control (MGAT4a: 87%, *p* = 0.008; MGAT5: 1.2-folds, *p* < 0.001) subjects, mirroring the HG-CD147 protein patterns (Figure 1E,F). 

**Figure 2:**
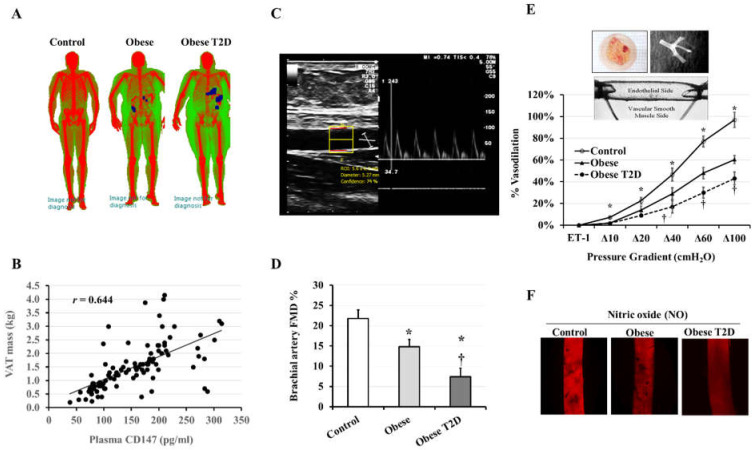
Anthropometric and vascular measurements in the study subjects. (**A**) Representative images of DEXA scanning performed for body composition assessment in obese, obese T2D, and non-obese subjects. (**B**) A scatter plot of plasma CD147 and total VAT mass measured via DEXA scanning. (**C**) Duplex B-mode and PWD (pulsed wave Doppler) ultrasound of brachial artery FMD. (**D**) Brachial artery FMD in obese, obese T2D, and control individuals was computed by deducting the average diameter at baseline from the largest measures after the blood pressure cuff was deflated. (**E**) FID measures in VAT-isolated arterioles correspond to intraluminal pressure gradients gradually increasing from Δ10 to Δ100 cmH_2_O. The insert images represent VAT tissues, isolated arterioles, and cannulated arterioles. (**F**) Representative images of NO production by VAT arterioles measured by fluorescence microscopy. Results represent the means ± standard error (SE). The * symbol indicates a *p*-value < 0.05 when comparing obese subjects with controls. The † symbol indicates a *p*-value < 0.05 when comparing obese and obese T2D subjects.

MMP2/9 activity in VAT-isolated proteins was evaluated as a function of the observed induction in CD147 glycosylation. The average MMP2/9 activity was higher in obese T2D participants than in controls (2.6 folds, *p* < 0.001) and obese non-diabetic subjects (1.2 folds, *p* = 0.042). When compared to controls, the obese non-diabetic group had a greater average of MMP2/9 activity (68% higher), but this increase was not statistically significant due to the large inter-individual variation (Figure 1G). In summary, these findings indicate higher levels of CD147 glycosylation and MMP activity in the VAT of obese T2D subjects compared to the obese non-diabetic and controls. Interestingly, there was a robust positive correlation between the mass of VAT measured via DEXA scanning and plasma CD147 (*r* = 0.644, *p* < 0.0001) (Figure 2A,B) as well as VAT measured HG-CD147 (*r* = 0.511, *p* < 0.001) and MMP activity (*r* = 0.321, *p* < 0.05). 

### 3.3. Vascular Measurements

The brachial artery FMD was measured using pulsed wave Doppler ultrasound (Figure 2C). The brachial artery diameter did not differ significantly among the three groups at baseline. The percentage of FMD increase in response to reactive hyperemia, on the other hand, was lower in obese T2D participants than in controls (1.6-folds, *p* < 0.0001) and obese non-diabetic subjects (46%, *p* = 0.028) (Figure 2D). Pulse wave velocity (PWV), which reflects arterial stiffness, was also measured in the study subjects using applanation tonometry. The obese T2D group had the highest PWV (12.9 ± 0.4) compared to obese non-diabetic subjects (10.8 ± 0.2, *p* = 0.114) and healthy controls (9.7 ± 1.2, *p* = 0.007). Across all pressure gradients (Δ10–Δ100 cmH_2_O), the FID observed in isolated VAT arterioles was greater in non-obese subjects than obese people (Figure 2E). The FID measurements in obese T2D were significantly lower than those in obese non-diabetic subjects, especially in response to higher pressure gradients (Δ40–Δ100 cmH_2_O). FID at Δ60 cmH_2_O, indicating the average physiological arteriolar pressure in the human body, was 1.6-fold (*p* < 0.0001) and 60% (*p* < 0.001) lower in obese T2D and obese non-diabetic subjects, respectively, compared to controls. Besides FID, flow-induced NO generation in VAT arterioles was assessed via a fluorescence emitting NO indicator (Figure 2F). The arteriolar NO generation was noticeably higher in the control group than in obese subjects. 

### 3.4. CD147 Correlations with Cardiometabolic Risk Factors

Plasma levels of CD147 were significantly correlated with several cardiovascular and metabolic risk factors (Table 2). After correction for multiple testing, plasma CD147 correlated positively with BMI, visceral fat mass, fasting plasma glucose (FPG), glycosylated hemoglobin, insulin resistance, and circulating markers of inflammation (CRP and IL6). Direct correlations were also identified between plasma CD147 and blood levels of carboxymethyl lysine (CML) and MMP2/9, as well as VAT levels of HG-CD147 and MGAT5. Direct correlations were seen between plasma CD147 and vascular parameters mainly arteriolar FID and flow-induced arteriolar NO production. Table 2 summarizes Pearson correlations (*r*) and *p* values for the correlation between plasma CD147 and measures of cardiometabolic risk. Multiple regression was performed to predict CD147 from BMI, body fat %, VAT mass, FPG, FPI, and HbA1c. These variables significantly predicted CD147, F (6, 113) = 39.966, *p* < 0.001, R2 = 0.503. All variables contributed significantly to the prediction, *p* < 0.001 (corrected *p* value). To test the association between plasma CD147 and vascular function, a second model of multiple regression analysis that included model 1 variables plus brachial FMD, PWV, and arteriolar FID was performed. Arteriolar FID (standardized coefficient β= −0.801, *p* < 0.0001) was significantly associated with CD147 after adjustment for other independent variables in this model. 

## 4. Discussion

Diabetes is characterized by hyperglycemia, which causes metabolic stress and cellular injury partly due to the generation of compounds such as AGEs [14,25]. Chronic hyperglycemia and AGE exposure alters protein glycosylation patterns, with glycosylated hemoglobin (HbA1c) being the most well-known example. Proteins, such as the MMP inducer CD147, have their activity determined by the degree of glycosylation [5]. Despite the well-established role of MMPs in inducing cardiovascular risk, the status of CD147 in diabetes and its link to vascular dysfunction has yet to be investigated. Accordingly, we sought to study CD147 in blood and its glycosylation status in visceral adipose tissues obtained from three groups of subjects representing the glucose tolerance continuum: healthy controls, obese non-diabetics, and obese with T2D. The study’s main conclusion is that circulating CD147 and its glycosylation levels in adipose tissues were significantly greater in obese T2D participants than in the other two groups. Despite the absence of statistical significance, obese non-diabetic participants had higher levels of glycosylated CD147 and glycosyltransferase enzymes than healthy controls. These levels were linked to impaired vascular function and increased cardiometabolic risk.

CD147 is a glycoprotein that exists in two forms: low glycosylated and high glycosylated. Previous research using various inhibitors of N-glycans revealed that CD147 glycosylation accounts for over 50% of the molecule’s size and strongly promotes its activity. CD147 glycosylation has been linked to important consequences in malignancies such as leukemia and liver cancer as well as the formation of atherosclerotic plaques [5,26]. These findings underscore the clinical importance of CD147 as a therapeutic target. The process of protein N-glycosylation depends on the availability of glucose and the activity of enzymes such as glycosidases and glycosyltransferases. This process is active and modifiable in response to changes in the extracellular environment, making protein glycosylation a reliable biomarker and an ideal therapeutic target. The N-glycosylation process begins with converting glucose to glucose-6-P, then glucose-1-P that interacts with UTP forming the glucose donor UDP-Glc. UDP-Glc participates in the first step in protein N-glycosylation [27]. Therefore, it is possible that the elevated glucose levels associated with T2D contribute to aberrant protein glycosylation. In addition to enzymatic glycosylation, non-enzymatic protein glycation occurs when reducing sugars are covalently attached to proteins, resulting in advanced glycation end products (AGEs). Protein glycosylation, whether enzymatic or non-enzymatic, was found to be induced in response to hyperglycemia. The role of hyperglycemia in promoting protein glycation has been reported in murine models by Hoffmann et al. [28] and was suggested to contribute to the advancement of diabetic complications. Furthermore, previous investigations, such as the Finland Cardiovascular Risk Study (FINRISK) [29] and the EPIC-Potsdam Cohort [30], corroborated these assumptions by highlighting the significance of enzymatic protein glycosylation as a biomarker of chronic hyperglycemia and predictor of diabetes-associated vascular dysfunction.

Due to its intrinsic glycosylation characteristics, CD147 is a candidate for perturbations caused by high blood glucose and AGEs accompanying diabetes and metabolic disorders. Therefore, investigating the expression and glycosylation of CD147 provides vital information into the genesis of diabetes comorbidities and how to address them therapeutically. Bao et al. [13] observed induction in CD147 glycosylation in immune cells exposed to high concentrations of glucose and AGEs, which was proposed to be mediated by a mechanism involving transforming growth factor bets (TGFβ) and tumor necrosis factor-alpha (TNFα). In our recently published work, we observed increases in CD147 expression and glycosylation in differentiated adipocytes in response to high glucose and CML treatment [12]. We also observed upregulations in the glycosyltransferase, MGAT5. In view of these findings, we sought to compare CD147 expression and glycosylation in adipose tissues from obese diabetics to obese non-diabetic and healthy subjects. The current study demonstrated, for the first time, different levels of the highly glycosylated (HG) form of CD147 protein as well as the relevant glycosyltransferases (MGAT4a and MGAT5) across the three groups, with obese T2D participants having the highest levels, followed by obese non-diabetics. Previous research has shown that CD147 detected on the plasma membrane and in cell culture media is the HG form, suggesting that glycosylation of CD147 is required for its translocation to the cell surface and subsequent extracellular release [31,32]. In support of these predictions, we observed a significant direct association between the HG form of CD147 in VAT and circulating levels of CD147; no equivalent correlation was seen for the LG form of CD147. These findings imply that circulating CD147 may represent CD147 status in adipose tissues and could be employed as a new biomarker for chronic hyperglycemia. However, further research with larger sample size is needed to support this assumption.

The significance of CD147 in cancer progression has long been established [33,34,35], and recently, the role of CD147 in mediating vascular and immune complications of severe acute respiratory syndrome coronavirus 2 (SARS-CoV-2) has piqued the interest of numerous research teams [36,37,38]. However, the role of CD147 in the development of diabetic complications has received less attention, and most of the current research is based on observations from rodent models. For example, a study by Xie et al. [39] tested the contributing role of CD147 glycosylation in neurovascular diabetic complications using a diabetic rat model. In this study, the authors reported higher levels of HG-CD147 in endothelial cells and astrocytes accompanied by higher MMP activity and blood-brain-barrier permeability in diabetic rats compared to non-diabetic ones. This profile has been linked to increased post-stroke hemorrhagic transformation and severe neurovascular consequences observed in diabetic rats. Another study by Wang et al. [40] reported higher CD147 glycosylation and MMP2/9 activity in cardiomyocytes from diabetic rats compared to non-diabetic controls, which accelerated cardiac pathological remodeling and the development of diabetic cardiomyopathy. On the clinical front, studies have shown that circulating CD147 can predict renal disease progression and impaired wound healing in T2D patients [41,42].

The current study is the first to show a robust relationship between circulating CD147 and standard cardiometabolic risk variables, including anthropometric and metabolic profiles on one side and augmented inflammation and vascular dysfunction on the other. Diabetes, and metabolic diseases in general, are characterized by a complicated interplay between inflammation and metabolic dysregulation, a process that includes multiple inflammatory mediators, and we believe that CD147 sits at the crossroads of this relationship. Previous mechanistic studies by our group and others pointed out the role of inflammation in promoting CD147 expression and glycosylation [12,13]. Considering the fact that diabetes is a chronic inflammatory condition with increased glucose substrate and enzymatic activity required for glycosylation, induced CD147 glycosylation would be an anticipated outcome that could result in a vicious circle of inflammation and disrupted metabolism. Nonetheless, future research should elucidate downstream signaling pathways activated by glycosylated CD147. In the current investigation, we focused on the link between glycosylated CD147 and enhanced MMP activity. However, alternative roles for CD147 have been proposed, including its function as a receptor for external inflammatory mediators, cyclophilins, and as a direct inducer of the transcription factor NFκB [6,41,42]. These alternatives were not investigated in the current research, and future research is required to elucidate them in the context of diabetes and vascular dysfunction.

Previous mechanistic studies have shown that glycosylation is an essential posttranslational modification for CD147 to promote MMP activity and that the latter was abolished by tunicamycin, an inhibitor of protein glycosylation [43,44]. Similar reductions in MMP activity were seen in our recent studies in adipocytes treated with tunicamycin or CD147 gene specific siRNAs [12], implying a role for glycosylated CD147. In the current study, MMP2/9 activity mirrored the HG CD147 protein abundance measured in the corresponding adipose tissue samples. In addition, we detected a positive relationship between the circulating factions of these proteins, CD147 and MMP2/9. MMPs are a class of zinc-containing enzymes that degrade the extracellular matrix and play an essential role in vascular remodeling. A rising body of evidence suggests that dysregulated MMPs play a role in cardiovascular disorders such as atherosclerosis, aneurysms, and hypertension [45]. MMPs were found in higher concentrations in atherosclerotic plaques, and patients with hypertension, myocardial infarction, and unstable angina had higher levels of circulating MMP2 and MMP9 than healthy people [46]. MMPs are also key players in inflammatory and oxidative stress pathways [47]. They are thought to represent a link between pathological states characterized by inflammation and the development of cardiovascular disease. Previous epidemiological research found a link between circulating MMPs and the risk of acquiring cardiovascular diseases [2,48], lending clinical significance to current research findings that motivate further exploration of CD147 as a prognostic biomarker and therapeutic target.

Vascular dysfunction is one of the earliest events in developing cardiovascular disease in obese and diabetic individuals [49]. In the current investigation, we assessed microvascular function ex vivo in isolated VAT arterioles, which is a robust approach because these arterioles are directly influenced by molecular alterations in the metabolically dysregulated milieu in adipose tissues. Multivariate regression analysis indicated that CD147 is a strong predictor of arteriolar FID after adjusting for other independent variables such as BMI, fat mass, and glucose metabolism parameters. CD147 was shown to have a similar association to other vascular parameters such as brachial FMD and PWV. This association with metabolic risk variables on one hand and vascular dysfunction on the other suggests that CD147 may play a role in the pathway that connects disordered metabolism and cardiovascular risk.

There are some strengths to the current study that should be highlighted. First, it is the first research to establish that adipose tissues in metabolically compromised people have increased CD147 glycosylation, which is associated with increased MMP activity. This effect is likely to produce tissue and vascular remodeling as well as enhanced inflammation locally or systemically if widespread tissues such as adipose tissues are involved. Targeting this mechanism may provide a viable strategy for MMP regulation, referring to CD147 as a possible therapeutic target in diabetes. Furthermore, this study discovered a dose-dependent pattern in the relationship between CD147 and critical cardiometabolic risk factors examined in three groups of participants who could represent the glucose tolerance continuum. Finally, unlike earlier studies that only evaluated vascular function in big conduit arteries like the brachial and femoral, we assessed FID in small resistance arterioles that are likely to be affected by VAT molecular alterations. Furthermore, small arterioles play an important role in peripheral resistance and blood pressure management, making this study therapeutically relevant and extremely impactful. Nevertheless, this study has some limitations. First, our sample size was small, which limited our statistical power to detect significant changes in various variables and their relationship with CD147. As a result, larger-scale investigations are needed to corroborate our findings and further comprehend the complex interaction between the variables evaluated. Second, the study’s cross-sectional design makes it difficult to draw causal inferences or suggest a specific direction for the association between tested variables. As a result, more longitudinal studies are needed to better understand the nature of these associations. Finally, unmeasured confounders or unknown factors, such as specific diets, medicines, supplements, or other inflammatory illnesses or comorbidities, could impact CD147 plasma levels. Even though we controlled for certain confounding variables in this study, numerous variables still need to be identified and investigated. One of the study’s major shortcomings is the lack of diabetic lean participants. According to recent epidemiological research, this group is not rare and is growing [50]. Furthermore, recent research has revealed that this group has a significant prevalence of cardiovascular morbidity [51]. As a result, incorporating this group in future studies might provide valuable insight into the interaction between obesity and diabetes in the development of vascular dysfunction.

## Figures and Tables

**Table 1:** Physical characteristics and cardiometabolic risk factors in the study subjects.

Variable	Healthy Controls (*n* = 40, ♂ = 18)	Obese (*n* = 40, ♂ = 15)	Obese T2D (*n* = 40, ♂ = 16)	*p* Value
Age, y	35.4 ± 1.3	36.1 ± 1.2	36.2 ± 1	0.8712
Weight, kg	74.4 ± 1.6	142.4 ± 3.8 *	144.4 ± 3.7 *	<0.0001
BMI, kg/m^2^	24.9 ± 0.5	48.7 ± 1.3 *	49.6 ± 1.1 *	<0.0001
WC, cm	91.5 ± 2.0	133.5 ± 3.2 *	135.5 ± 4 *	<0.0001
Body fat, %	32.2 ± 2.5	51.3 ± 1.1 *	54.3 ± 1.0 *	<0.0001
Body lean, %	65.3 ± 2.4	42.5 ± 0.7 *	38.5 ± 0.9 *	<0.0001
VAT mass, kg	0.7 ± 0.1	2.3 ± 0.3 *	2.7 ± 0.2 *	<0.0001
HR, bpm	74 ± 2	81 ± 1 *	84 ± 1 *	<0.0001
SBP, mmHg	118 ± 2	132 ± 4 *	141 ± 2 *	<0.0001
DBP, mmHg	75 ± 1	82 ± 1 *	87 ± 1 *†	<0.0001
FPG, mg/dL	87 ± 8	103 ± 9	135 ± 8 *†	0.0003
FPI, µU/mL	8.1 ± 0.8	11.2 ± 0.4 *	18.9 ± 1.2 *†	<0.0001
HOMA-IR	1.6 ± 0.1	2.5 ± 0.2 *	6.4 ± 0.4 *†	<0.0001
HbA1c, %	5.3 ± 0.1	5.4 ± 0.2	6.8 ± 0.3 *†	<0.0001
T-Chol, mg/dL	155 ± 9	165 ± 8	190 ± 4 *†	0.0031
HDL, mg/dL	56 ± 6	44 ± 2	34 ± 1 *	0.0003
LDL, mg/dL	81 ± 7	99 ± 4 *	131 ± 4 *†	<0.0001
TG, mg/dL	92 ± 11	108 ± 8	128 ± 8 *	0.0225
NO, µmol/L	6.0 ± 0.4	3.7 ± 0.2 *	2.7 ± 0.1 *†	<0.0001
CRP, mg/dL	0.7 ± 0.1	3.8 ± 0.2 *	4.1 ± 0.4 *	<0.0001
IL6, pg/mL	5.2 ± 0.6	15.6 ± 3.6 *	26.8 ± 1.6 *†	<0.0001
TNFα, pg/mL	2.6 ± 0.2	3.4 ± 0.1 *	5.7 ± 0.2 *†	<0.0001
CD147, pg/mL	87.7 ± 19.1	121.1 ± 21.6	184.1 ± 11.6 *†	0.0009
MMP2, ng/mL	6.1 ± 1.5	14.3 ± 2.4 *	18.1 ± 3.1 *	0.0023
MMP9, ng/mL	8.3 ± 2.5	19.8 ± 2.3 *	20.8 ± 4.6 *	0.0140
MG, ng/mL	38.1 ± 9.0	51.1 ± 13.1	89.3 ± 11.1 *†	0.0045
CML, ng/mL	81.2 ± 12.1	140.4 ± 25	211.5 ± 15.2 *†	<0.0001

Abbreviations: BMI, body mass index; WC, waist circumference; VAT, visceral adipose tissues; HR, heart rate; SBP, systolic blood pressure; DBP, diastolic blood pressure; HOMA-IR, homeostatic model assessment for insulin resistance; FPG, fasting plasma glucose; FPI, fasting plasma insulin; HbA1c, glycosylated hemoglobin; T-Chol, total cholesterol; HDL, high-density lipoprotein; LDL, low-density lipoprotein; TG, triglycerides; NO, nitric oxide; CRP, C-reactive protein; IL6, interleukin 6; TNFα, tumor necrosis factor-alpha; MMP, matrix metalloproteinase; MG, methylglyoxal; CML, carboxymethyl lysine. The * symbol indicates a *p*-value < 0.05 when comparing obese or obese diabetics with controls and the † symbol indicates a *p*-value < 0.05 when comparing obese and obese T2D subjects using an appropriate post hoc test.

**Table 2:** Correlations between plasma CD147 and cardiometabolic risk factors.

Variable	Correlation Coefficient	*p*-Value
Weight	0.329	0.0002
BMI	0.360	<0.0001
VAT mass	0.644	<0.0001
FPG	0.480	<0.0001
HOMA-IR	0.410	<0.0001
HbA1c	0.320	0.0004
Plasma CRP	0.365	<0.0001
Plasma IL6	0.420	<0.0001
Plasma CML	0.451	<0.0001
Plasma MMP2	0.366	<0.0001
Plasma MMP9	0.436	<0.0001
Fat HG-CD147	0.662	<0.0001
Fat MGAT5	0.447	<0.0001
Arteriolar FID	−0.305	0.0007
Arteriolar NO	−0.281	0.0018

Abbreviations: BMI, body mass index; VAT, visceral adipose tissues; FPG, fasting plasma glucose; HOMA-IR, homeostatic model assessment for insulin resistance; HbA1c, glycosylated hemoglo-bin; CRP, C-reactive protein; IL6, interleukin 6; CML, carboxymethyl lysine; MMP, matrix met-alloproteinase; HG, Highly glycosylated; MGAT5, alpha-1,6-mannosylglycoprotein 6-beta-N-acetylglucosaminyltransferase; FID, flow-induced dilation at Δ60; NO, nitric oxide.

## Data Availability

The data presented in this study are available in the article.
